# Conserved epitopes with high HLA-I population coverage are targets of CD8^+^ T cells associated with high IFN-γ responses against all dengue virus serotypes

**DOI:** 10.1038/s41598-020-77565-2

**Published:** 2020-11-24

**Authors:** Thiruni N. Adikari, Francesca Di Giallonardo, Preston Leung, Alba Grifoni, Alex Sette, Daniela Weiskopf, Rowena A. Bull, Fabio Luciani

**Affiliations:** 1 Immunogenomics Laboratory, Kirby Institute for Infection and Immunity, University of New South Wales, Sydney, Australia; 2 School of Medical Sciences, University of New South Wales, Sydney, Australia; 3 Division of Vaccine Discovery, La Jolla Institute for Immunology, La Jolla, CA USA; 4 Department of Medicine, University of California San Diego, La Jolla, CA USA

**Keywords:** Infectious diseases, Immunology, Computational biology and bioinformatics, Genome informatics, Phylogeny

## Abstract

Cytotoxic CD8^+^ T cells are key for immune protection against viral infections. The breadth and cross-reactivity of these responses are important against rapidly mutating RNA viruses, such as dengue (DENV), yet how viral diversity affect T cell responses and their cross-reactivity against multiple variants of the virus remains poorly defined. In this study, an integrated analysis was performed to map experimentally validated CD8^+^ T cell epitopes onto the distribution of DENV genome sequences across the 4 serotypes worldwide. Despite the higher viral diversity observed within HLA-I restricted epitopes, mapping of 609 experimentally validated epitopes sequences on 3985 full-length viral genomes revealed 19 highly conserved epitopes across the four serotypes within the immunogenic regions of NS3, NS4B and NS5. These conserved epitopes were associated with a higher magnitude of IFN-γ response when compared to non-conserved epitopes and were restricted to 13 HLA class I genotypes, hence providing high coverage among human populations. Phylogeographic analyses showed that these epitopes are largely conserved in most of the endemic regions of the world, and with only some of these epitopes presenting distinct mutated variants circulating in South America and Asia.This study provides evidence for the existence of highly immunogenic and conserved epitopes across serotypes, which may impact design of new universal T-cell-inducing vaccine candidates that minimise detrimental effects of viral diversification and at the same time induce responses to a broad human population.

## Introduction

Dengue virus (DENV) has become a major public health problem placing half the global population at risk with an estimated 390 million annual infections worldwide^1^. The incidence of DENV infection has increased 30 fold during recent decades^2^ posing an alarming risk on human health. A disease which was only restricted to nine countries prior to 1970, has now become endemic in more than 125 countries in both tropics and subtropics with 70% of the cases occurring in Asia^3^. The majority of DENV infections are asymptomatic, and only a small proportion of cases develops more severe forms including dengue hemorrhagic fever (DHF) and dengue shock syndrome^4,5^. To date, there is no effective vaccine or antiviral therapy for dengue and treatment is limited to supportive management.

DENV is a single-stranded positive RNA virus with a genome size of 10.7 kb consisting of three structural (Capsid, Precursor membrane protein, Envelope) and seven non-structural proteins (NS1, NS2A, NS2B, NS3, NS4A, NS4B, NS5)^6^. Like other RNA viruses, high genome replication errors result in extensive genetic diversity within each serotype^7^. Four serotypes (DENV1-4) are known, that share 65–75% sequence homology with each other^8^. It is known that humoral adaptive response is strongly associated to clearance of infection from one serotype, which also provides long lasting immunity against that particular serotype^9^, but limited cross-protection against the remaining serotypes^10^. The substantial genomic diversity across the four DENV serotypes poses a challenge in the identification of immune targets across conserved epitopes which can provide protection against multiple infections.

The role of cellular immune responses in determining clearance of DENV infections and long-term immunity has been investigated, however the exact contribution of T cells to immune protection remains unclear^11,12^. Cytotoxic CD8^+^ T cells are known to be associated to clearance, via the production of pro-inflammatory cytokines such as IFN-γ and TNF-α, while CD4^+^ T cells have been found to contribute to enhancement of B and CD8^+^ T-cell responses. In DENV infections, both CD4^+^ and CD8^+^ T cells have shown to generate memory phenotype following clearance^13,14^. Several studies from human infections^15–17^ and animal models^18–20^ have demonstrated a protective role for CD8^+^ T cell responses. CD8^+^ T cell responses targeting several serotype specific as well as cross-reactive antigenic epitopes have been reported with cytotoxic activity in acute phase of DENV infections^21,22^. Similarly, T cell responses targeting non-structural regions has been detected with functional activity measured by IFN-γ production after DENV vaccination^23,24^. Furthermore, high magnitude and poly-functional CD8^+^ T cell responses, i.e., those that upon stimulation have degranulation capacity as well as interleukin secretions (e.g., IFN-γ, TNF-α, and IL-2) have been found to be associated with protection from DENV infections^15,19^. In addition, several class I HLA alleles, e.g. the HLA-I B*35:01 and A*33:01, have been identified conferring protection against DENV infections^15,25,26^.

Despite these findings, the exact contribution of dengue specific CD8^+^ T cell responses to immunological protection from re-infection remains unclear. For instance, there is limited knowledge on the memory CD8^+^ T cell responses that can be recalled during a secondary infection, and whether these reactivated cells are functional and contribute to protection. It has been postulated that antigenic sin may somewhat limit the functional response of memory reactivated cells against a secondary infection, however these theories have been mostly based on humoral responses^27^. Only a limited number of studies investigated the frequency of epitope-specific CD8^+^ T cells that occur during primary infection and how these contribute to the control or elimination of secondary infections. Notably, the limited studies so far were based on a small sample size and a limited number of epitopes which often were serotype specific^28,29^. Therefore, better characterization of the specificity of CD8^+^ T cell responses and the degree of diversification within epitope targets need to be further elucidated.

In this study, we have employed a bioinformatics approach to estimate viral diversity from a large set of full-length viral genome sequences, and to quantify the relationship between viral diversity within epitope targets and the magnitude of CD8^+^ T cells against these targets, utilizing 609 experimentally validated epitopes and also IFN-γ ELISPOT responses from two independent large cohort studies on healthy previously exposed subjects. With these data we discovered a subset of epitopes within conserved regions of the DENV genome that were associated to a high magnitude of IFN-γ production and that were restricted to a broad range of HLA-I genotypes, thus providing high population coverage.

## Materials and methods

### Selection of DENV full genome sequences

A total of 3985 full-length DENV sequences were retrieved from Virus Pathogen Database and Analysis Resource (www.viprbrc.org, accessed in August 2018). These sequences were 1736 DENV-1, 1241 DENV-2, 816 DENV-3, and 192 DENV-4 specific and covering a 60 years time-period. The number of sequences varied across serotypes and countries. All the sequences were aligned using the Geneious aligner in the Geneious-Prime v2019.0.3) (http://www.geneious.com)^30^.

### Phylogenetic analysis

DENV nucleotide sequences for full length coding regions were aligned using MAFFT, implementing the L-INS-I algorithm^31^. Phylogenetic trees were estimated for each serotype separately using RAxML (version 8.2.11), employing the GTR + Γ nucleotide substitution model and 100 bootstrapping replicates^32^.

### Analysis of genomic diversity

The diversity of the DENV genomes was measured using Shannon entropy (SE) calculated from the distribution of non-synonymous and synonymous single nucleotide variants (SNV) using a bioinformatics pipeline as previously described^33^. SE values were calculated from the distribution of SNVs with a frequency of occurrence 0.1% within the sequences analysed. SE was measured per genomic region and per amino acid position across the DENV to determine both intra-serotype and inter-serotype variability, respectively. SE values were normalised by the length of the genomic region, and these values were utilised to calculate the mean values within sub-genomic regions and also within and outside epitope segments. These values were calculated for the near full‐length ORF as well as for each viral protein coding region (Core, PrM, E, NS1, NS2A, NS3, NS4A, NS4B, NS5) separately.

### Selection of conserved HLA-I restricted epitopes

Experimentally validated epitopes eliciting antigen specific CD8^+^ T cell responses were retrieved from the Immune Epitope Data Base (www.iedb.org, accessed in August 2018). A total of 609 experimentally validated MHC class I epitopes (157 DENV1, 279 DENV2, 77 DENV3 and 96 DENV4) were found with a positive IFN-γ ELISPOT assay from one or more independent experiment. The selection criteria for conserved epitopes shown in Table 1 were: (i) experimentally tested with positive response in all 4 serotypes from the IEDB data base; (ii) a maximum of 2 amino acid mutations between the epitope sequences across the four serotypes. Anchor positions of these epitopes were retrieved from the peptide-MHC binding motif data from IEDB resources and published data^34–38^.

**Table 1:** Summary of viral diversity within and outside epitope regions.

	DENV1	DENV2	DENV3	DENV4
Number of HLA-I restricted epitopes	157	279	77	96
Proportion of the genome covered by epitopes	33.10	49.57	17.17	25.79
Number and % of non-synonymous SNV within epitope regions	79 (7.0%)	142 (8.7%)	43 (7.3%)	80 (9.2%)
Number and % of non-synonymous SNV outside epitope regions	203 (8.9%)	175 (10.5%)	175 (6.2%)	242 (9.6%)

### Analysis of IFN-γ ELISPOT responses

The SFC values were retrieved from two previous publications^15,39^, reporting population-based studies from Sri Lanka and Nicaragua (see “Results” for details). The background threshold for the spot forming cell (SFC) values per subject was 20 SFC/10^6^ PBMCs, as per the original publication. Analysis was performed using the average response (SFC/10^6^ PBMC) per positive donor (Total SFC/pos donors). If the same epitope was tested in both studies with positive SFC responses, then the mean value was considered.

### Population HLA coverage

Allele frequencies across human populations were retrieved from the online data base www.allelefrequencies.net. The allele frequency was calculated as the total number of copies of the allele in the population (alleles/2n) in decimal format. When multiple HLA population coverage were available within a region e.g. across more than one country within that region of interest, then an average value was considered for each HLA type and used it as the estimate of the coverage for that specific region.

### Statistical analysis

Comparative analyses between Shannon entropy distributions as well as IFN-γ responses between conserved and serotype specific epitopes were performed using a Mann–Whitney U test for unpaired data. Graphs were generated using GraphPad Prism version 7.0 (GraphPad, San Diego, CA).

## Results

### Analysis of the diversity of DENV genome revealed heterogenous distribution of mutations in all four serotypes

**Figure 1:**
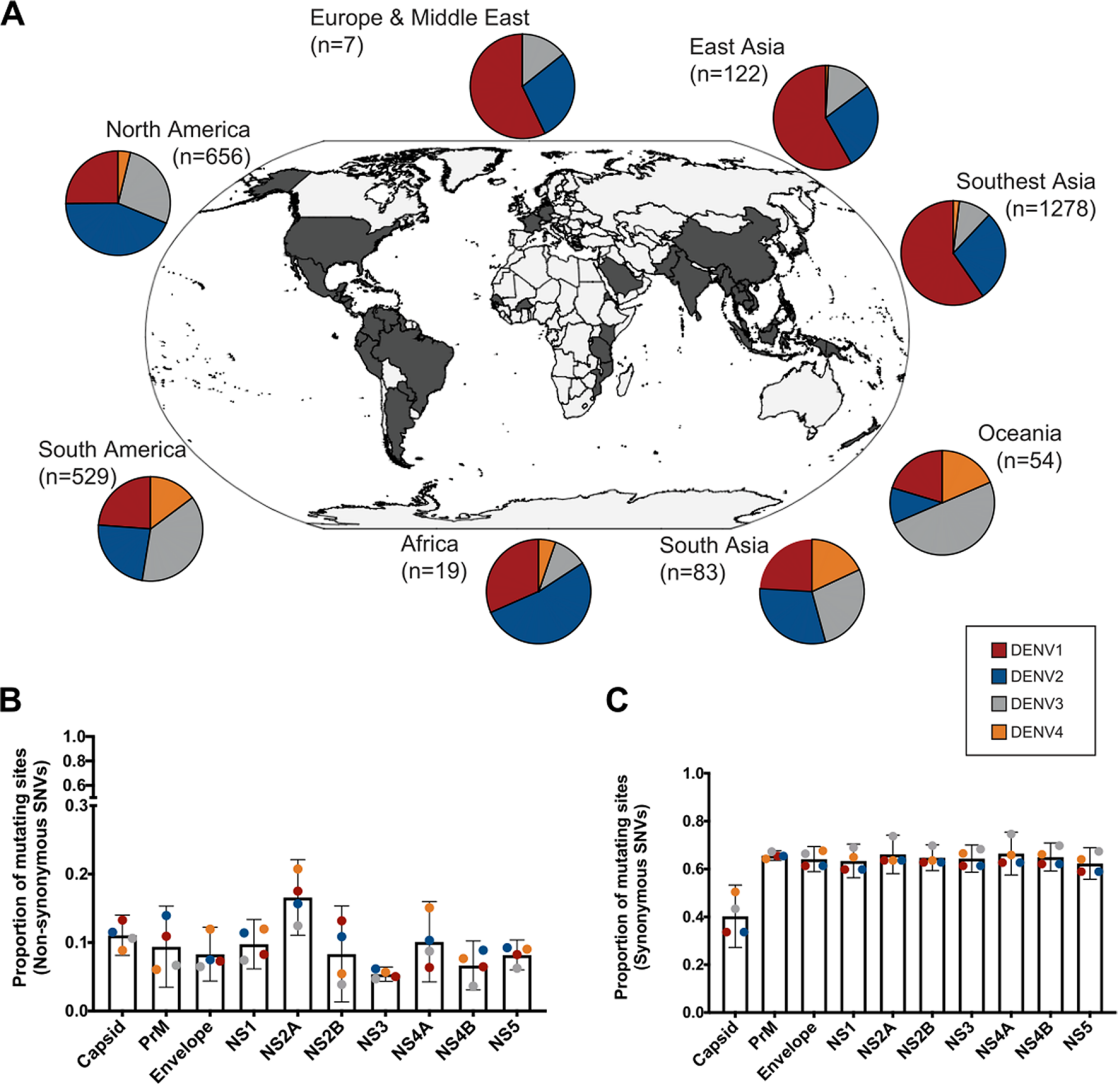
World-wide distribution of DENV serotypes and genomic diversity. (**A**) Geographic distribution of the full-length DENV genome sequences utilised in this study. (**B**) Proportion of mutating sites with non-synonymous (**B**) and synonymous (**C**) SNVs for each serotype and within each sub-genomic region.

To identify the level of genomic diversification across the four DENV serotypes circulating worldwide, a total of 3985 full-length DENV genomes (DENV1-1736, DENV2-1241, DENV3-816, DENV4-192) were analysed, representing a wide geographical area across the globe. The majority of these sequences (76%) were sampled from Asia. 59.6% of DENV1 sequences were sampled from Southeast Asia while 58.8% of DENV2 from South America and Central/North America (Fig. 1A). The sequences were obtained from samples collected over a period of 60 years, the oldest being a DENV2 sequence from Papua New Guinea in 1944 and the latest from the recent 2019 DENV1 epidemic in Mexico, and with approximately 80% of sequences derived from samples collected between 2005 and 2015. The majority of the regions represented in this analysis had available sequences across all four serotypes, with the exception of Europe and Middle East, and with DENV4 the least represented, reflecting the lower prevalence compared to other serotypes (Supplementary Table S1).

As sub-genomic regions of the genome are different in size, the proportion of genomic sites carrying synonymous and non-synonymous single nucleotide variants (SNVs) were calculated for each sub-genomic region. Synonymous SNVs accounted for the majority of the genomic diversity, with more than half of the sites presenting synonymous SNV for each serotype (DENV1-59.2%, DENV2-67%, DENV3-50%, DENV4-63.5%, Fig. 1C). Non-synonymous SNVs were found in 10% or less of the sites for each serotype (9.6%, 10.5%, 7%, 10% respectively, Fig. 1B). The smallest proportions of synonymous SNVs were within the Capsid, while NS2A revealed the highest. Conserved regions in terms of non-synonymous SNVs were identified within E, NS2B, NS3, NS4B, and NS5.

**Figure 2:**
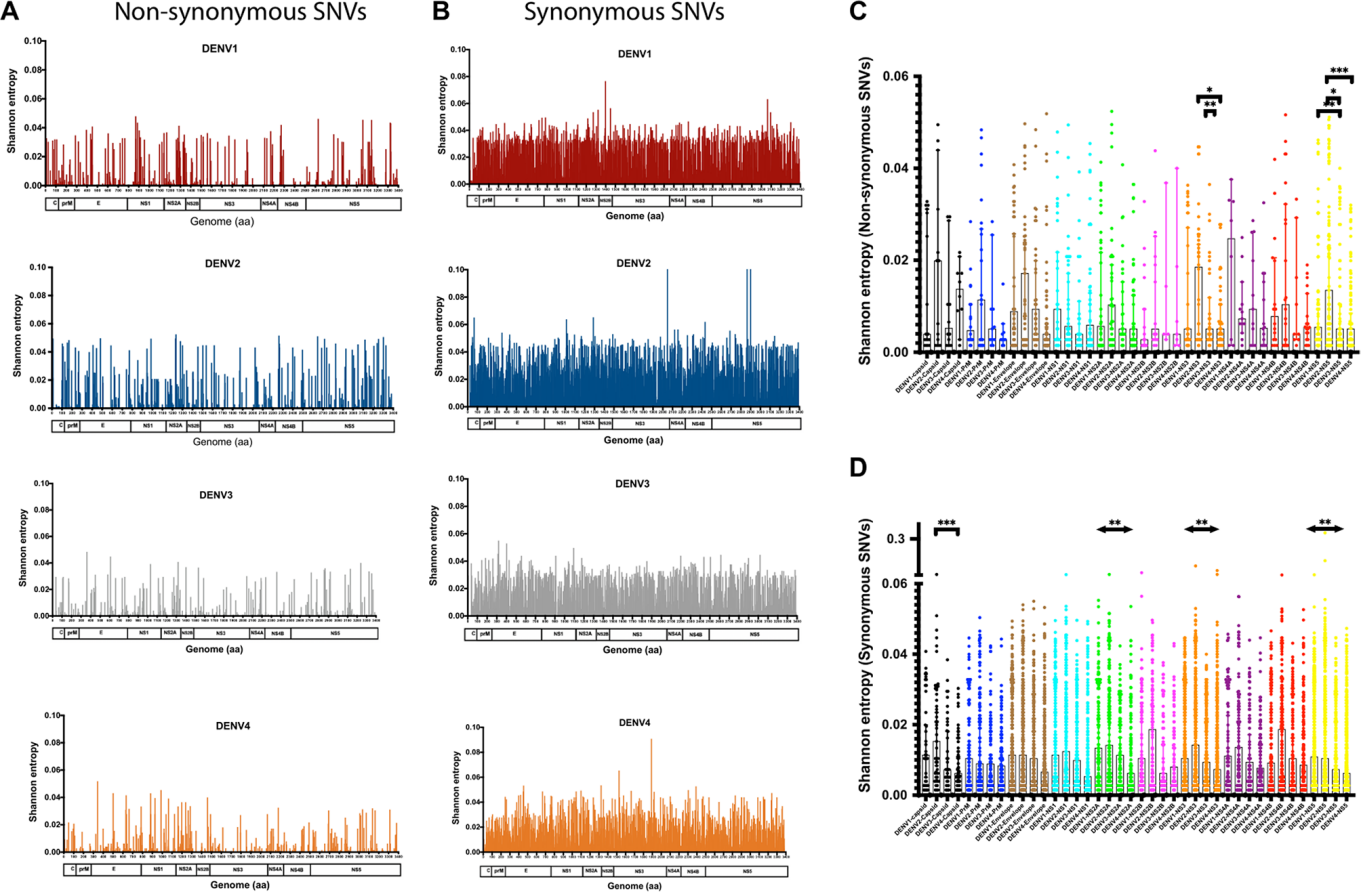
Viral diversity analysis of DENV genomes. Viral diversity using Shannon entropy measures from the distribution of SNVs across the four serotypes from non-synonymous (**A**) and synonymous (**B**). Analysis within sub-genomic regions utilizing non-synonymous (**C**) and synonymous (**D**) SNVs across the four serotypes revealed significant differences within NS3 and NS5. Statistical tests performed using non-parametric Mann–Whitney U test. Shannon entropy values are colour-coded by serotype.

To further identify conserved regions within the genome, Shannon entropy (SE) per position was calculated, which is a measure of viral diversity and can be used to identify patterns of mutations across each serotype. A higher SE value indicates high genomic variability, while a SE value of zero corresponds to an invariant genomic site. Overall, the distribution of SE values corresponding to non-synonymous and synonymous SNVs were heterogeneously distributed along the genome (Fig. 2A,B and significant differences were found in the distribution of SE values for both synonymous and non-synonymous SNVs within the NS3 and NS5 regions (Fig. 2C,D). Further analysis of the distribution of SE values considering only the Asian sequences—which accounted for approximately 60% of the total sequences respectively—showed similar patterns to the ones observed from the full data sets (Supplementary Fig. S1).

### Immunodominant CTL epitopes exist within regions conserved across all four DENV serotypes

**Figure 3:**
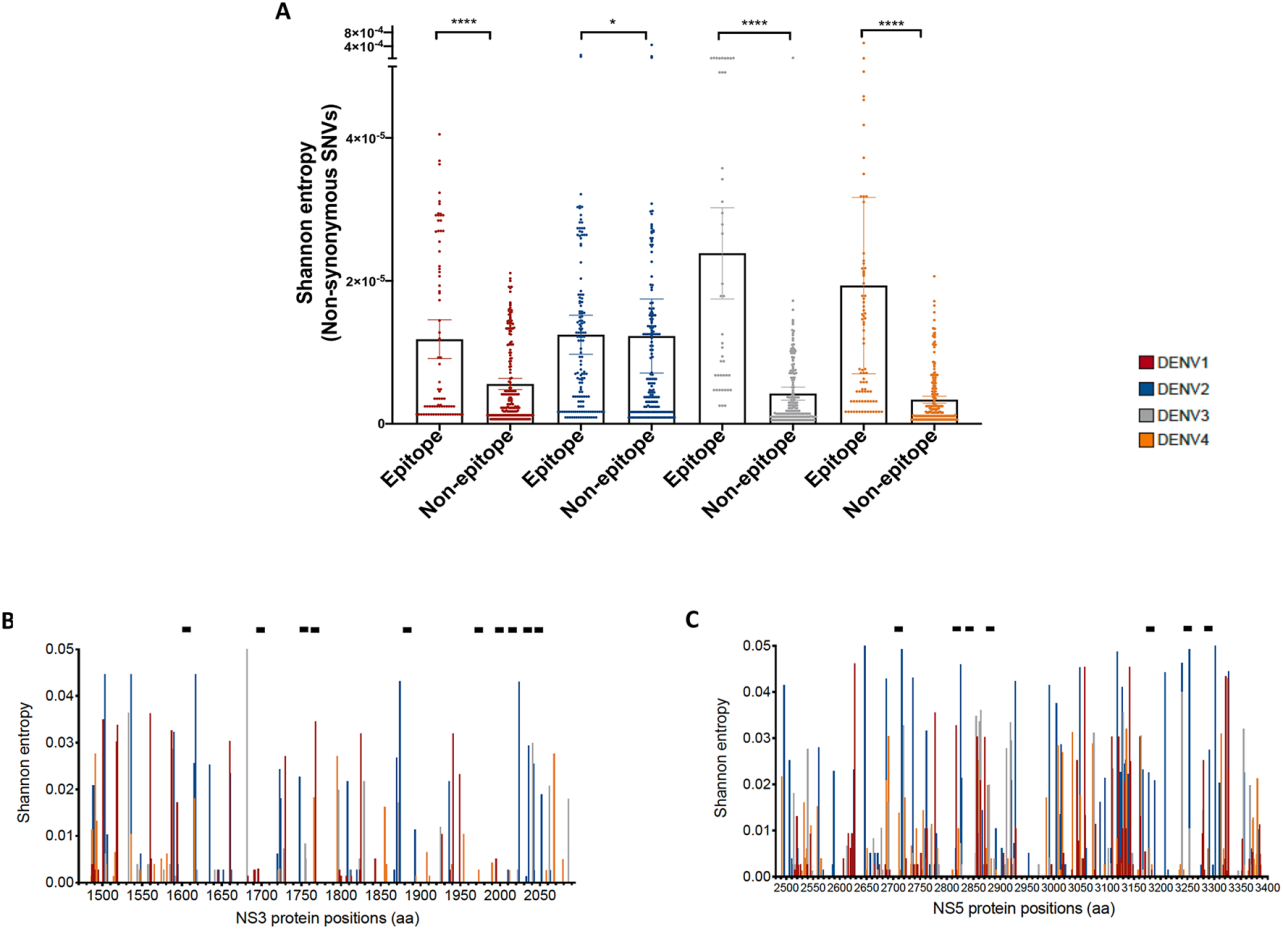
Viral diversity analysis revealed conserved regions within the NS3 and NS5 regions. (**A**) Distribution of Shannon entropy values within and outside epitope regions using non-synonymous SNVs. Statistical tests performed using non-parametric Mann–Whitney U test. Shannon entropy values are colour-coded by serotype. (**B**,**C**) Distribution of Shannon entropy values from non-synonymous SNVs within NS3 and the NS5 regions. Black bars represent the location of the selected epitopes within conserved regions of the NS3 and the NS5 segments.

To assess the relationship between the distribution of epitopes targeted by CD8^+^ T cells and the overall viral diversity, SE values from the distribution of non-synonymous SNVs between epitope and non-epitope regions were analysed across the full-length genomes. For this analysis, 609 experimentally validated HLA class I epitopes were retrieved from the Immune Epitope database (IEDB) (Supplementary Table S2), and mapped onto the genomes, which revealed immunogenic regions within NS3 and NS5. The majority of these epitopes were DENV1 and DENV2 specific, covering 33.1% and 49.6% of the genome respectively, and about a fifth of the genome for both DENV3 and DENV4 (Table 1). The distribution of SE (normalized by epitope length) from non-synonymous SNVs revealed higher values within epitope regions across all four serotypes when compared to non-epitope regions (Mann–Whitney p < 0.05 Fig. 3A, Supplementary Table S3).

Despite the increased diversity within HLA-I epitopes, 19 conserved epitope regions were discovered, that differed only by 0, 1 or 2 amino acids away from the DENV2 consensus sequence (Table 2). All of the conserved epitopes were independently validated from one or more studies via positive IFN-γ ELISPOT tests, and of these 2 were within the NS4B and the remaining 17 within the NS3, and NS5 regions (Fig. 3 B and C, Table 2). Four of these 19 epitopes were 100% conserved across the four serotypes, and two more epitopes were 100% conserved with at least 2 other serotypes. These 19 epitopes were restricted across 13 HLA types, comprising of 6 HLA-A and 7 HLA-B subtypes. Since anchor positions within the epitope play an integral part for determining effective presentation, we also assessed whether the epitope variants carried amino acid mutations in these anchor positions. This analysis revealed none of the epitope regions carried mutations within anchor positions, thus suggesting that epitope variants may have similar antigen presentation efficiency compared to wild type.

**Table 2:** Characteristics of the conserved epitopes.

No	Epitope ^a^	Serotype	Region	Position	HLA restriction	Mutations in anchor	% (no)of donors responded ^b^	Average (Total SFC/positive donors)^b^	No. of studies with positive response
1	GTSGSPI**VN**R	1	NS3	1608–1617	HLA-A 11:01	No	Positive	Positive	13
	GTSGSPIIDR	2			HLA-A 11:01		3.2 (2/63)	226.7	9
	GTSGSPII**N**R	3			HLA-A 11:01		4.5 (1/22)	373.3	9
	GTSGSPII**N**R	4			HLA-A 11:01		Positive	Positive	3
2	APTRVVA**S**EM	1	NS3	1697–1706	HLA-B*07:02	No	8.75 (7/80)	111.1	6
	APTRVVAAEM	2			HLA-B*35:01		14.9 (7/47)	167.6	2
	APTRVVAAEM	3			HLA-B*35:01		3 (1/33)	393.33	4
	APTRVVAAEM	4			HLA-B*07:02		1.8 (1/56)	450	1
3	VPNYN**M**IIM	1	NS3	1750–1758	HLA-B*35:01	No	3(2/66)	54.2	1
	VPNYNLIIM	2			HLA-B*35:01, B*07:02		3(1/33)	385	4
	VPNYNLIIM	3			HLA-B*35:01, B*07:02		Positive	Positive	4
	VPNYNLI**V**M	4			HLA-B*35:01		Positive	Positive	1
4	DPASIAARGY	1	NS3	1765–1774	HLA-B*35:01	No	10.1 (9/89)	184.4	1
	DPASIAARGY	2 3			HLA-B*35:01		Positive	Positive	1
	DPASIAARGY				HLA-B*35:01		Positive	Positive	1
	DP**S**S**V**AARGY	4			HLA-B*35:01		Positive	Positive	1
5	DISEMGANF	1	NS3	1885–1893	HLA-A*26:01	No	Positive	Positive	1
	DISEMGANF	2			HLA-A*26:01		Positive	Positive	1
	DISEMGANF	3			HLA-A*26:01		Positive	Positive	1
	DISEMGANF	4			HLA-A*26:01		Positive	Positive	1
6	TPEGIIP**AL**F	1	NS3	1975–1984	HLA-B*35:01	No	19.1 (17/89)	226.9	3
	TPEGIIPSMF	2			HLA-B*35:01		20.2 (18/89)	373.83	8
	TPEGIIP**AL**F	3			HLA-B*35:01		4.3 (2/47)	165	1
	TPEGIIP**TL**F	4			HLA-B*35:01		18.0 (16/89)	256	6
7	GEARKTFV**E**L	1	NS3	2002–2011	HLA-B*40:01	No	4.1 (4/96)	377.5	2
	GEARKTFVDL	2			HLA-B*40:01		4.1 (4/96)	372.5	2
	GE**S**RKTFV**E**L	3			HLA-B*40:01		3.2 (2/63)	546.7	4
	GE**Q**RKTFV**E**L	4			HLA-B*40:01		3.2 (2/63)	510.8	1
8	LPVWL**S**YKV	1	NS3	2017–2025	HLA-B*51:01	No	3 (1/33)	313.33	1
	LPVWLAYKV	2			HLA-B*51:01		3 (1/33)	230	2
	LPVWLA**H**KV	3			HLA-B*51:01		Positive	Positive	1
	LPVWLSYKV	4			HLA-B*51:01		Positive	Positive	1
9	**Q**Y**S**DRRWCF	1	NS3	2031–2040	HLA-A*24:02	No	1.4 (1/72)	30	4
	NYADRRWCF	2			HLA-A*24:02		Positive	Positive	4
	**K**Y**T**DRKWCF	3			HLA-A*24:02		Positive	Positive	1
	**S**Y**K**DREWCF	4			HLA-A*24:02		Positive	Positive	1
10	LEENM**D**VEIW	1	NS3	2048–2057	HLA-B*44:03	No	11.1 (1/9)	23.33	1
	LEENVEVEIW	2			HLA-B*44:03		7.1 (1/14)	23.33	2
	LEENM**D**VEIW	3			HLA-B*44:03		Positive	Positive	1
	LEENMEVEIW	4			HLA-B*44:02		Positive	Positive	2
11	**H**PASAWTLY	1	NS4B	2276–2284	HLLA-B*35:01	No	3.4 (3/89)	48.33	3
	RPASAWTLY	2			HLLA-B*07:02		4.3 (2/47)	264.17	4
	**H**PASAWTLY	3			HLLA-B*35:01		Positive	Positive	4
	RPASAWTLY	4			HLLA-B*07:02		Positive	Positive	3
12	ATGP**LT**TLW	1	NS4B	2442–2450	HLA-B*58:01	No	5.6 (2/36)	315	1
	ATGPISTLW	2			HLA-B*58:01		5.6 (2/36)	117.5	1
	ATGPI**T**TLW	3			HLA-B*58:01		5.6 (2/36)	450	1
	ATGPI**L**TLW	4			HLA-B*58:01		5.6 (2/36)	469.2	1
13	SRNSTHEMY	2	NS5	2701–2709	HLA-A*02:01	No	Positive	Positive	1
	SRNSTHEMY	1			HLA-A*02:01		Positive	Positive	1
	SRNSTHEMY	3			HLA-A*02:01		Positive	Positive	1
	SRNSTHEMY	4			HLA-A*02:01		Positive	Positive	1
14	KPWDV**I**PMV	1	NS5	2821–2829	HLA-B*51:01, B*55:01	No	6.1 (2/33)	373.3	1
	KPWDVVPMV	2			HLA-B*51:01		6.1 (2/33)	275.83	1
	KPWDVVPMV	3			HLA-B*55:01		Positive	Positive	1
	KPWDV**I**PMV	4			HLA-B*51:01, B*55:01		Positive	Positive	1
15	DTTPFGQQR	1	NS5	2836–2844	HLA-A*33:01, A*68:01	No	17.9 (5/28)	486.7	3
	DTTPFGQQR	2			HLA-A*33:01, A*68:01		22.7(5/22)	489.9	3
	DTTPFGQQR	3			HLA-A*33:01, A*68:01		Positive	Positive	3
	DTTPFGQQR	4			HLA-A*33:01, A*68:01		Positive	Positive	3
16	**K**PR**I**CTREEF	1	NS5	2881–2890	HLA-B*07:02	No	6.77 (4/59)	274.72	3
	TPRMCTREEF	2			HLA-B*07:02		4.3 (2/47)	190.8	9
	**K**PR**L**CTREEF	3			HLA-B*07:02		10.2 (6/59)	275.7	5
	**N**PR**L**CTREEF	4			HLA-B*07:02		8.5 (5/59)	323.5	2
17	KVRKDI**P**QW	1	NS5	3177–3185	HLA-B*57:01	No	Positive	Positive	2
	KVRKDIQQW	2			HLA-B*57:01		Positive	Positive	1
	KVRKDI**P**QW	3			HLA-B*57:01		Positive	Positive	2
	KVRKDI**P**QW	4			HLA-B*57:01		Positive	Positive	2
18	ETACLGKSY	1	NS5	3246–3254	HLA*26:01	No	33.33(5/15)	285.9	1
	ETACLGKSY	2			HLA*26:01		Positive	Positive	1
	ETACLGKSY	3			HLA*26:01		Positive	Positive	1
	ETACLGKSY	4			HLA*26:01		Positive	Positive	1
19	MTTEDML**S**VW	1	NS5	3295–3304	HLA-B*58:01	No	Positive	Positive	1
	MTTEDMLTVW	2			HLA-B*58:01		Positive	Positive	1
	MTTEDML**T**VW	3			HLA-B*58:01		Positive	Positive	1
	MTTEDML**K**VW	4			HLA-B*58:01		Positive	Positive	1

^a^Mutations from the DENV2 variant are in bold.

^b^Data from the Sri Lanka^15^ or the Nicaragua^39^ cohort studies.

### Conserved epitopes are associated with high magnitude of IFN-γ CD8^+^ T cell responses

**Figure 4:**
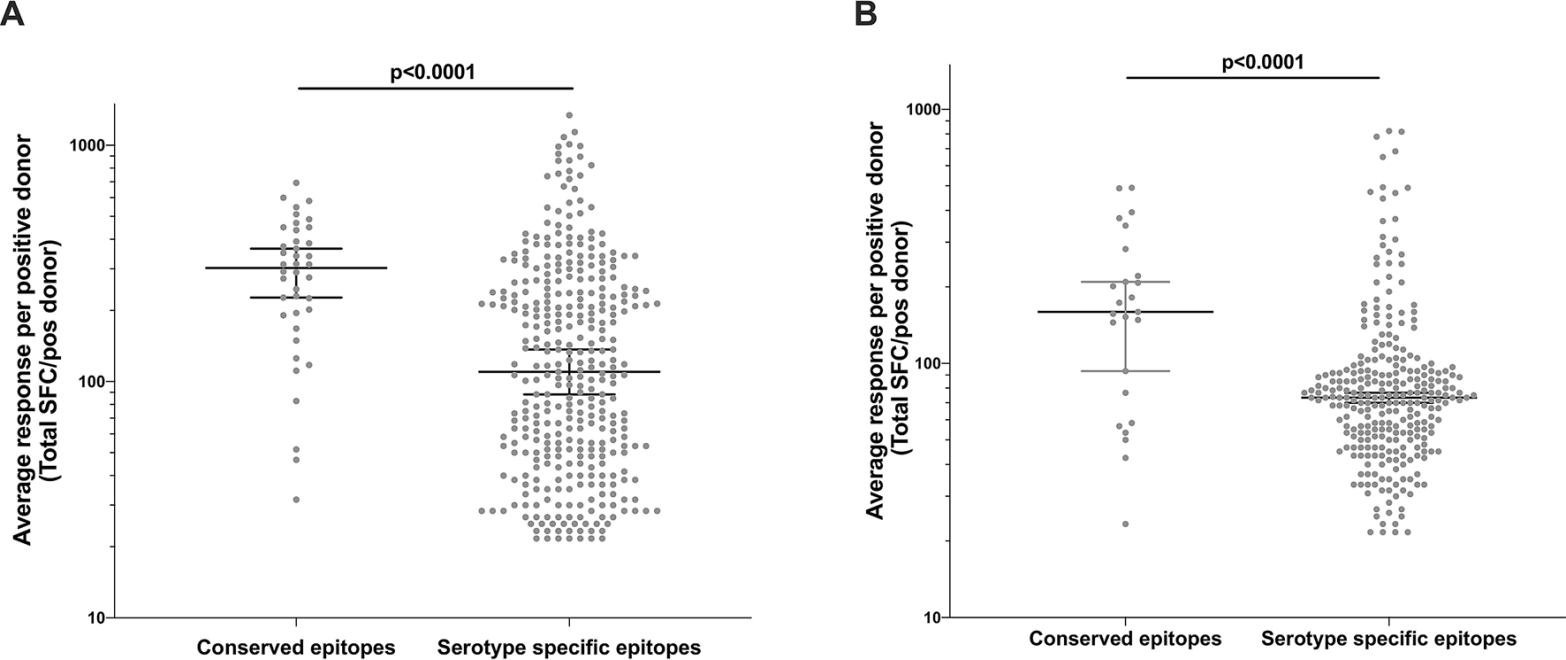
High magnitude of CD8^+^ T cell responses against conserved epitopes. (**A**) Comparison of the magnitude of IFN-γ responses from CD8^+^ T cells targeting highly conserved epitopes, and serotype specific epitopes identified in the Sri Lanka (**A**) and Nicaragua (**B**) datasets. Shown are dot plots with Spot Forming Counts (SFC)/10^6^ PBMCs normalized by the number of subjects with a positive response against the targeted epitope. Horizontal bars represent median values with 95% confidence intervals.

To assess the magnitude of the CD8^+^ T cell responses targeting the conserved epitope regions, IFN-γ ELISPOT data were retrieved from two cohort studies carried out in Sri Lanka and Nicaragua^15,39^, which comprise 699 IFN-γ responses across 357 subjects. In the Sri Lanka study, there were 40 IFN-γ responses (from a total of 397 responses across 227 positive subjects) associated with conserved epitopes (median 302.7 total SFC per positive donor). These responses were significantly higher when compared to those targeting serotype specific epitopes (n = 357, median = 110.0 total SFC per positive donor) (Mann Whitney U test, p < 0.0001, Fig. 4A). Similarly, the analysis of 302 IFN-γ responses across 130 positively tested subjects from the Nicaragua cohort revealed 25 highly conserved epitopes responses associated with high IFN-γ production (Mann Whitney U test, p < 0.0001, median = 159.6 total SFC per positive donor) compared to serotype specific responses (n = 277, median = 73.33 total SFC per positive donor) (Fig. 4B). About 70% of the epitopes with positive IFN-γ response that were identified in the Nicaragua study matched the epitope tested in the Sri Lankan study and 12 of the 19 epitopes in Table 2 were present in both of these datasets. The HLA-B*35:01 restricted responses were the most frequent in both the populations. The top 8 epitopes with the highest IFN-γ responses (900 to 1339 total SFC per positive donor in Sri Lanka, 820–490 SFC in Nicaragua) were observed in serotype specific epitopes and restricted to HLA-B*40:01 and HLA-B*57:01 in Sri Lanka and to HLA-B*44:03, and HLA-A*26:01 in Nicaragua data sets. Each of these large serotype specific IFN-γ responses however were detected in only one patient from a relatively large number of total donors tested (N = 35–90). There were IFN-γ responses available for each serotype specific variants within five epitope regions revealed that responses against DENV2 epitopes sequences were higher in three of these five epitopes, and all encoded in the NS3 region (Supplementary Fig. S2).

### Phylogenetic analysis revealed minimal bias towards geographical regions with regards to prevalence of conserved epitopes

**Figure 5:**
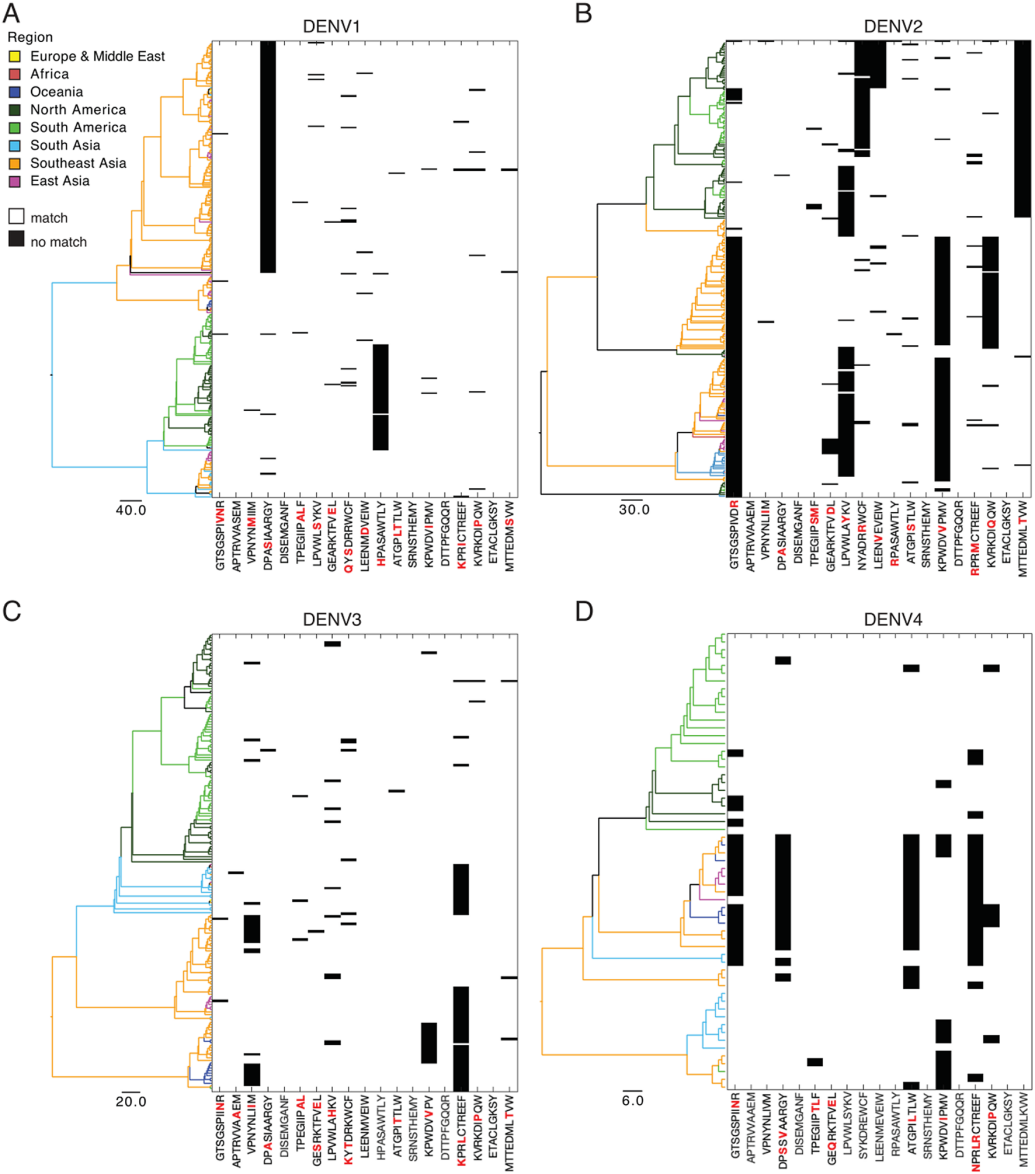
Geographic distribution of conserved epitopes. Phylogenetic trees of full-length genomes along with heat maps showing the distribution of conserved epitope regions and variants in all major geographic regions. Branch length indicates nucleotide substitutions per site, and these are coloured according to geographic regions. Epitope variants are shown in black and specific amino acid changes are marked in red.

Amino acid differences within the epitope sequences of the 19 conserved regions were mapped to phylogenetic trees generated from the full length DENV genomes. This analysis revealed that only a few amino acid variants were specifically circulating in distinct geographic regions, while the majority of the epitopes were relatively conserved across the world (Fig. 5). Notably, DENV2 specific epitope sequences were the most variable, and with four epitopes showing single amino acid variants dominating in Asia while three other variants were the most prevalent in the Americas. In contrast, for DENV3 there were no major amino acid variants associated with specific regions of the world. For DENV1 only one of the 19 epitope sequences had mutated sequences differing between Southeast Asia and the Americas, while the remaining epitopes were relatively conserved. For DENV4 sequences, four epitopes showed region-specific mutational patterns. These results suggest that the majority of the T cell epitopes remain largely conserved across the world.

### Conserved epitopes are restricted by a broad range of HLA alleles across ethnicities

The investigation of the distribution of HLA-restrictions of the 19 epitopes revealed a broad HLA coverage among human populations, with an average worldwide coverage of 60% and 41% for the HLA-A and HLA-B, respectively (Table 3). Within all Asian ethnicities (East, South-East and South Asia), the average HLA coverages were 78% and 45% for HLA-A and HLA-B alleles, respectively. The lowest coverage observed was for African populations with 25.8% and 28.1% respectively. Six of the 19 epitopes were restricted to HLA B*35:01 and A*33:01, which are known to be associated with protection from disease in humans^15^. Twelve out of the 19 conserved epitopes were presented by HLA-B alleles, which is consistent with the larger diversity of HLA-B types among human populations compared to HLA–A types. The analysis also revealed three alleles, i.e., HLA-A*02:01, A*11:01 and A*24:02, which were among the most prevalent in Asia and Oceania.

**Table 3:** Population coverage of the HLA restrictions identified from the conserved epitopes.

HLA-I Restriction	No of epitopes	Genomic region	SE Asia (%)	South Asia (%)	East Asia (%)	South America (%)	North America (%)	Oceania (%)	Europe (%)	Africa (%)	Middle East (%)
A*02:01	1	NS4B	20	13	29	32.2	24.4	10.6	29	13.9	28.9
A*11:01	1	NS3	28	14	22	4.5	4	15.3	5.6	0.1	2
A*24:02	1	NS3	21.4	15	22	13.6	12.8	34	9.6	0.7	8
A*26:01	2	NS3	2	3.6	3.8	2.2	2.9	2.2	4	2.1	10
A*33:01	1	NS5	11	14	10.5	2.7	3.2	1.3	1.3	1.0	5.0
A*68:01	1	NS5	0.4	3.6	0.6	8.7	7.2	1.3	4.0	7.0	10
B*07:02	3	NS3,NS5	4.1	3	3	5.6	6.7	3.5	6	3.4	8.1
B*35:01	5	NS3,NS5	3.5	9.3	8.1	19.5	17.8	2.3	7.4	9	7
B*40:01	1	NS3	15	12	16.6	5.7	7	17	6	0.9	1.5
B*44:03	1	NS3	3.5	7.1	1.1	9.1	6.7	7.4	14	2.8	2.8
B*51:01	2	NS3,NS5	4.1	10	6.1	7.0	4.4	2.2	6.8	4	12
B*55:02	1	NS5	2.6	2.4	1.6	0.9	0.8	4.8	1.7	-	-
B*58:01	2	NS4B,NS5	6.7	7.4	6.9	1.6	2.0	1.2	1.1	8.0	4
Total HLA-A			82.8	63.2	87.9	63.9	54.5	64.7	53.5	24.8	63.9
Total HLA-B			39.5	51.2	43.4	49.4	45.4	38.4	43	28.1	35.4

## Discussion

This study revealed the presence of highly conserved HLA-I restricted epitopes across all four serotypes that elicited a stronger IFN-γ response compared to serotype specific T cell responses. Despite the surprisingly higher viral diversity within epitope regions across the four serotypes, this analysis revealed highly conserved epitopes that were presented by a broad range of HLA alleles, including some with known associations to protection. These results provide further evidence for the existence of cross-serotype viral targets for CD8^+^ T cell responses that elicit strong IFN-γ response and can be further investigated in future designs of vaccine targets to increase response against a broad range of viral strains and yet maintaining broad population coverage.

Limited data exist on the magnitude and breadth of CD8^+^ T cell responses that are retained from primary infections and utilised as memory recall in secondary responses^11,40^. Previous studies that investigated the extent of conservation of DENV genome and its effect on the T cell responses were performed only with a limited number of full-length sequences, which could significantly impact and overestimate the presence of cross-serotype responses^41–43^. In this study, the higher IFN-γ responses identified with CD8^+^ T cells targeting conserved epitopes across all four DENV serotypes may be the result of repeated exposure to DENV infections across different serotypes, which result in the selection and expansion of T cell responses that are recalled from precedent infection if the viral targets remain conserved. This is indeed supported by the fact that the majority of subjects from the Sri Lanka and Nicaragua cohorts investigated in this study were healthy adult donors, thus likely to have been exposed to more than one infection during their lives. High magnitude of CD8^+^ T cell responses is also consistent with the hypothesis that high-fitness costs are associated with mutations within conserved regions of the genome, which impairs viral replication or transmission of the mutated strains, as it has been already demonstrated for HIV^15,44,45^ and hepatitis C virus^46,47^. Impaired fitness of the virus may explain the evolutionary adaptation of the virus as a result of strong IFN-γ response against those regions of the genome. For instance, studies on influenza virus revealed the existence of several epitopes targeted by CD8^+^ T cells that remain conserved across strains, thus suggesting that this conserved set of epitopes could be exploited for vaccine targets^48^.

The presence of conserved regions within NS3 and NS5 is consistent with previous findings which were limited to a smaller data set^45,49^, and is supported by recent findings using genome-wide mutagenesis, which revealed that these two genomic regions along with prM and NS2A are intolerant to insertional mutagenesis^50^. However, the striking observation of high viral diversity within epitope regions suggest ongoing adaptation of the virus to the host T cell response. Therefore, it is conceivable that during future epidemics, immunogenic epitope regions may eventually acquire novel variants, within these conserved regions, thus impacting viral recognition.

The conserved epitopes identified in this study could be specifically tested for their role in inducing a strong cross-reactive response, thus suggesting that antigenic sin may be an important contribution, rather than limitation to immune protection. These epitopes may be also useful for designing T-cell based vaccine targets that can minimise the effect of viral diversification and at the same time cover large human populations. Recent results with Dengvaxia vaccine trials, which lack non-structural dengue specific genome regions in its construct, have not reported significant success, rather an association with severe disease^51^. In contrast, recent work on Takeda vaccine TAK3, which contains non-structural regions of the DENV2 has shown efficacy across all serotypes^52^, and notably, this vaccine has been also tested for level of T cell responses, showing high level of IFN-γ production across serotypes^53^. A previous study also showed that vaccination with tetravalent live attenuated vaccine induce CD8^+^ T cell responses against conserved epitopes of the DENV genome^24^. Notably, in this study only 7% of the total epitopes identified was serotype specific, and the majority were conserved across all four serotypes within one amino acid. It should be noted that this previous study however considered only a limited number of DENV sequences, and a maximum of 10 sequences per geographic region, hence it is likely that many of the conserved epitopes identified carry major variants in other population.

The observation of strong IFN-γ responses against multiple conserved epitopes restricted by HLA-B 35:01 and A-33*01 is consistent with previous findings of high frequency and magnitude of T cell responses restricted by these alleles^15^, which are also known to be associated with protection^25,54^. Even though there are no data supporting how the cellular immune responses against DENV vary according to different ethnic groups, the higher heterogeneity of HLA-B compared to HLA-A alleles^55^, could limit the population coverage of T-cell-inducing vaccines, as in the case of African ethnicities, which have the highest genetic diversity among human populations^56^. Notably, the presence of strong CD8^+^ T cell responses against conserved regions of the viral genome and targeted by specific HLA has been previously reported for other viruses, e.g. in HIV^43^ and in influenza virus^57^. Overall, further studies should investigate in more details the strength of T cell responses against conserved regions of the DENV genomes, and specifically investigate the set of HLA restrictions in endangered communities with rare HLA types, such as indigenous populations.

There are limitations of this study that should be acknowledged. Firstly, the degree of conservation of DENV genome could be evolving due to rapid adaptation and consequent escape from host natural responses. Due to viral genomes evolving during epidemics, conserved genomic regions may eventually mutate following host responses. Secondly, this study was based on a set of more than 600 experimentally validated epitopes. Other epitopes may exist, for instance in exposed individuals during acute phase of infection, which are difficult to identify after viral clearance due to low precursor frequencies in the blood. These responses could become low frequency in the blood post clearance and remain within lymph-node and skin, which is the major site of DENV specific immune response. Future studies should investigate the extent of poly-functional responses targeting conserved epitopes soon after acute infection or in vaccine trials.

In conclusion, this study provided strong evidence that cross-reactive CD8^+^ T cell responses play a role in controlling infections and be robust to viral diversity. A better understanding of T cell responses in current vaccine trials and natural responses may be helpful to better understand protection, while cellular responses can be integrated with neutralizing and non-neutralizing B cell responses to achieve high level of protection across human populations.

## Supplementary information

Supplementary Table 1.

Supplementary Table 2.

Supplementary Table 3.

Supplementary Figure 1.

Supplementary Figure 2.

Supplementary Figure Legends.
