# Supplementary material for: Conserved epitopes with high HLA-I population coverage are targets of CD8+ T cells associated with high IFN-γ responses against all dengue virus serotypes
Source: Sci Rep. 2020 Nov 24;10:20497. doi: 10.1038/s41598-020-77565-2 (PMC7687909; doi:10.1038/s41598-020-77565-2)
Supplement: Supplementary file 1 — Supplementary Table 1. [file 41598_2020_77565_MOESM1_ESM.docx]

Supplementary Material

**Conserved epitopes with high HLA-I population coverage are targets of CD8^+^ T cells associated with high IFN-γ responses against all dengue virus serotypes**

Thiruni N Adikari^1,2^, Francesca Di Giallonardo^1^, Preston Leung^1,2^, Alba Grifoni^3^, Alex Sette^3,4^, Daniela Weiskopf^3^, Rowena A Bull^2^ and Fabio Luciani^1,2*^

^1^Immunogenomics Laboratory, The Kirby Institute, University of New South Wales, Sydney, Australia

^2^ School of Medical Sciences, University of New South Wales, Sydney, Australia

^3^Division of Vaccine Discovery, La Jolla Institute for Immunology, La Jolla, CA, United States

^4^Department of Medicine, University of California San Diego, La Jolla, CA, United States

**Supplementary Table 1**: Geographic distribution of the full-length DENV genome sequences analysed in this study.

| Serotype | World | Asian | % of Asian sequences | Origin of Sequences |
| --- | --- | --- | --- | --- |
| DENV1 | 1736 | 1315 | 76% | Brazil, Kenya, USA, Nicaragua, Venezuela, Colombia, Puerto Rico, Mexico, Virgin Islands, Jamaica, Belize, Dominican Republic, Honduras, Guatemala, Peru, Cuba, East Timor, Burkina Faso, Tanzania, Ecuador, Papua New Guinea, Saudi Arabia, Australia, France, St Kitts and Nevis, Vietnam, Cambodia, Japan, Philippines, Singapore, India, China, Pakistan, Sri Lanka, Thailand, Indonesia, South Korea, Myanmar, Haiti |
| DENV2 | 1241 | 604 | 49% | Brazil, Kenya, USA, Nicaragua, Venezuela, Colombia, Puerto Rico, Mexico, Virgin Islands, Jamaica, Belize, Dominican Republic, Honduras, Guatemala, Peru, Cuba, East Timor, Burkina Faso, Tanzania, Ecuador, Papua New Guinea, Saudi Arabia, Australia, France, Haiti, Vietnam, Cambodia, Philippines, Singapore, India, China, Pakistan, Sri Lanka, Thailand, Indonesia, Taiwan, South Korea, Myanmar, Laos, Malaysia |
| DENV3 | 816 | 232 | 28% | USA, Venezuela, Colombia, Brazil, New Caledonia, French Polynesia, Wallis and Futuna, Ecuador, Papua New Guinea, Senegal, Mozambique, Anguilla, Saint Lucia, Peru, Guyana, Cooks Islands, Grenada, Saudi Arabia, Cuba, Timor Leste, Vietnam, Cambodia, Philippines, Singapore, India, China, Pakistan, Sri Lanka, Thailand, Indonesia, Taiwan, South Korea, Myanmar, Laos, Malaysia |
| DENV4 | 192 | 43 | 22% | USA, Venezuela, Colombia, Brazil, New Caledonia, French Polynesia. Wallis and Futuna, Ecuador, Papua New Guinea, Senegal, Cambodia, Philippines, Singapore, India, China, Pakistan, Sri Lanka, Thailand, Indonesia, Japan |
