# Supplementary figures and images for: Conserved epitopes with high HLA-I population coverage are targets of CD8+ T cells associated with high IFN-γ responses against all dengue virus serotypes
Source: Sci Rep. 2020 Nov 24;10:20497. doi: 10.1038/s41598-020-77565-2 (PMC7687909; doi:10.1038/s41598-020-77565-2)

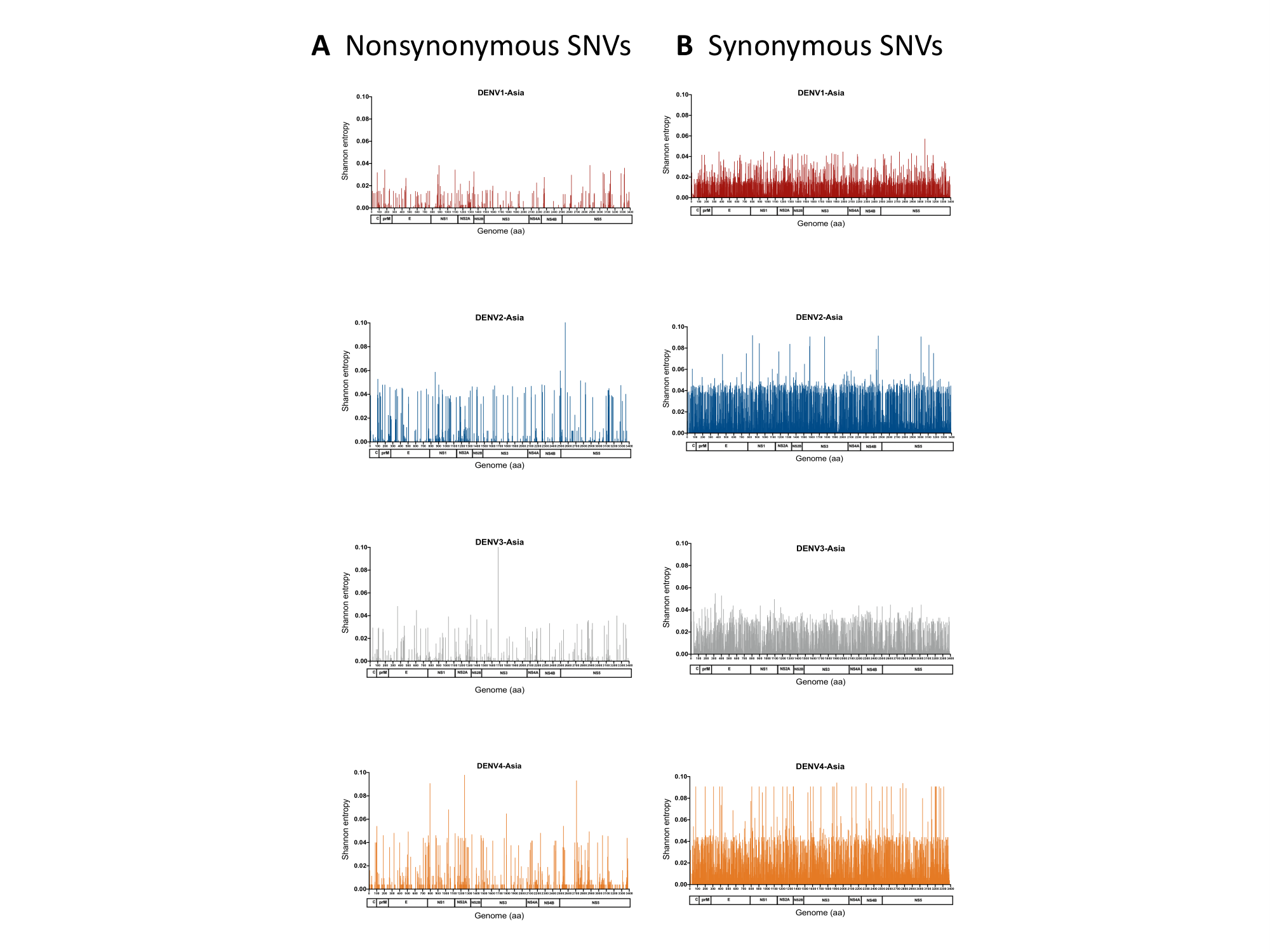

Supplement: Supplementary file 4 — Supplementary Figure 1. [file 41598_2020_77565_MOESM4_ESM.tiff]

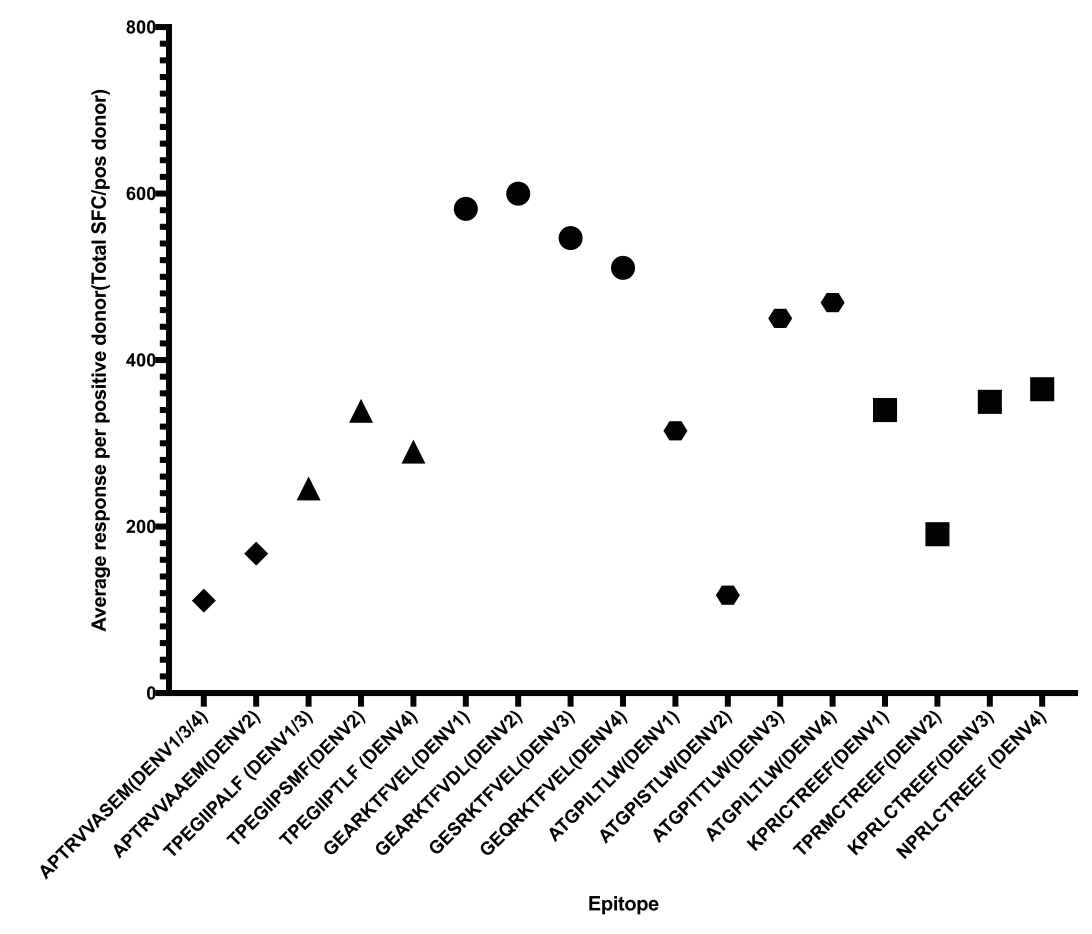

Supplement: Supplementary file 5 — Supplementary Figure 2. [file 41598_2020_77565_MOESM5_ESM.tiff]
