# Supplementary material for: Conserved epitopes with high HLA-I population coverage are targets of CD8+ T cells associated with high IFN-γ responses against all dengue virus serotypes
Source: Sci Rep. 2020 Nov 24;10:20497. doi: 10.1038/s41598-020-77565-2 (PMC7687909; doi:10.1038/s41598-020-77565-2)
Supplement: Supplementary file 6 — Supplementary Figure Legends. [file 41598_2020_77565_MOESM6_ESM.docx]

**Supplementary Figure 1.** Viral diversity using Shannon entropy measures from the distribution of SNVs across the four serotypes from non-synonymous (A) and synonymous (B) using sequences from Asia.

**Supplementary Figure 2.** IFN-﻿γ responses measured in SFC against serotype specific variants of five conserved epitopes.
